# DNA-based taxonomy of a mangrove-associated community of fishes in Southeast Asia

**DOI:** 10.1038/s41598-021-97324-1

**Published:** 2021-09-07

**Authors:** Danial Hariz Zainal Abidin, Siti Azizah Mohd. Nor, Sébastien Lavoué, Masazurah A. Rahim, Noorul Azliana Jamaludin, Noor Adelyna Mohammed Akib

**Affiliations:** 1 Centre for Global Sustainability Studies (CGSS), Level 5, Hamzah Sendut Library, Universiti Sains Malaysia, 11800 Penang, Malaysia; 2 School of Biological Sciences, Universiti Sains Malaysia, 11800 Penang, Malaysia; 3 Institute of Marine Biotechnology, Universiti Malaysia Terengganu, 21030 Kuala Terengganu, Terengganu Malaysia; 4 Fisheries Research Institute, 11960 Batu Maung, Penang Malaysia; 5 Fisheries Research Institute, Kampung Acheh, 32000 Sitiawan, Perak Malaysia

**Keywords:** Biodiversity, Conservation biology, PCR-based techniques

## Abstract

The Merbok Estuary comprises one of the largest remaining mangrove forests in Peninsular Malaysia. Its value is significant as it provides important services to local and global communities. It also offers a unique opportunity to study the structure and functioning of mangrove ecosystems. However, its biodiversity is still partially inventoried, limiting its research value. A recent checklist based on morphological examination, reported 138 fish species residing, frequenting or subject to entering the Merbok Estuary. In this work, we reassessed the fish diversity of the Merbok Estuary by DNA barcoding 350 specimens assignable to 134 species initially identified based on morphology. Our results consistently revealed the presence of 139 Molecular Operational Taxonomic Units (MOTUs). 123 of them are congruent with morphology-based species delimitation (one species = one MOTU). In two cases, two morphological species share the same MOTU (two species = one MOTU), while we unveiled cryptic diversity (i.e. COI-based genetic variability > 2%) within seven other species (one species = two MOTUs), calling for further taxonomic investigations. This study provides a comprehensive core-list of fish taxa in Merbok Estuary, demonstrating the advantages of combining morphological and molecular evidence to describe diverse but still poorly studied tropical fish communities. It also delivers a large DNA reference collection for brackish fishes occurring in this region which will facilitate further biodiversity-oriented research studies and management activities.

## Introduction

Estuaries and coastal wetlands which feature mangrove ecosystem are transition zones that link terrestrial and freshwater habitats with the sea^1^. Mangrove ecosystem delivers essential ecosystem services, including shoreline protection, nutrient production and fisheries resources. In consequence, mangrove ecosystem plays a vital role in supporting local communities’ socio-economic pursuits^2^. Unfortunately, such crucial human-nature relationship is threatened by habitat pollution, destruction, and overfishing^3^. It is also impacted by other factors such as species invasion, and climate change^1^.

The less disturbed tropical estuaries, especially their mangrove area, generally harbour rich, unique and complex faunal communities^4^, combining the presence of salinity-tolerant resident species along with regularly or occasionally frequenters. Frequenters include mainly marine species, which use this ecosystem either to feed, shelter, breed, or nurse their young^5^. Inventorying and monitoring biodiversity in these ecosystems is primordial for long term sustainability because biodiversity ensures stability and resistance towards any disturbance or potential invasion through complex species-species interactions^1^. However, biodiversity is still poorly documented in many mangrove ecosystems, particularly those of Southeast Asia, which hampers further research on their functioning and management.

**Figure 1:**
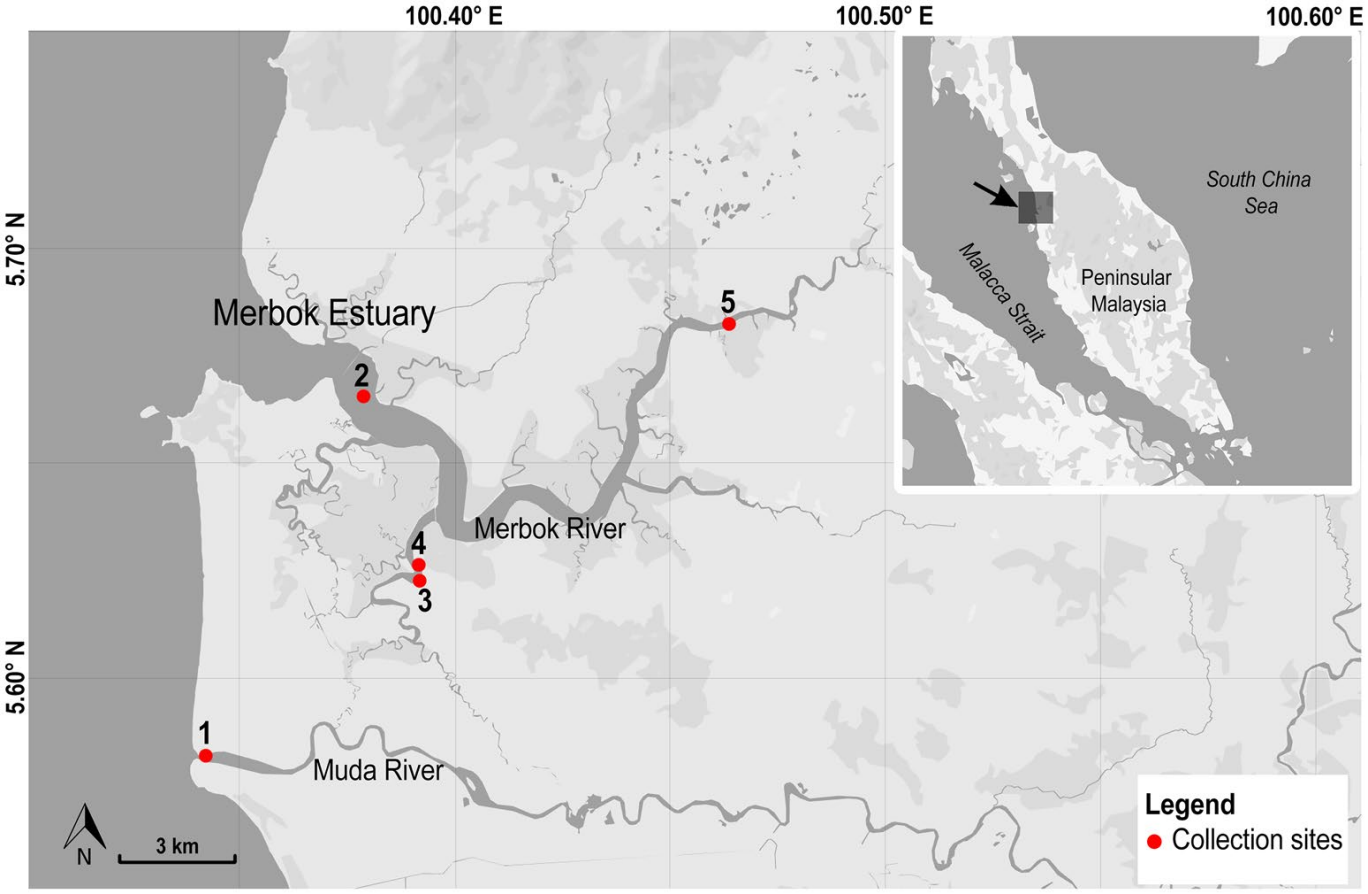
Sampling localities across the study area, which covers the Merbok Estuary (Merbok River) and Muda River. Sampling sites; 1: Kuala Muda Whispering Market, 2: Pompang Sungai Merbok, 3 and 4: Pompang Batu Lintang, 5: Semeling Bridge. Inset map shows the location of the study area within Peninsular Malaysia. Maps are generated using QGIS v.3.4.11 and edited in Adobe Photoshop CC 2019.

Malaysia is part of the Sundaland biodiversity hotspot, which is recognized for its astounding levels of diversity and endemism^6,7^. Considering only fishes, ^8^ reported the presence of a total of 1418 marine and brackish species in Malaysian waters, occupying various coastal habitats, including the threatened mangrove ecosystems^9^. One of the largest remaining intact patches of mangrove forests is located within the Merbok Estuary, north-west Peninsular Malaysia, facing the Strait of Malacca (Fig. 1). The estuary was gazetted as a permanent forest reserve, the Sungai Merbok Mangrove Forest Reserve in 1951, and is the second largest mangrove forest in Peninsular Malaysia after the Larut Matang Forest Reserve. The Merbok Estuary and its surroundings constitute a dynamic and productive ecosystem, which supports the World’s highest mangrove species diversity per unit area within a contiguous habitat, with 39 of the estimated 70 true mangroves species described globally^10^. This area also represents important resource grounds for local populations^11,12^.

Due to its biological, ecological, and socio-economic importance, the Merbok Estuary has been the focus of research during the last two decades, including some biodiversity inventories (trees and gastropods^13^; shrimps^11^; fishes^12,14^; mangrove trees^10^). The latest ichthyological survey has inventoried 138 fish species from 47 families in the estuary and adjacent marine environment, revealing a rich fish fauna^15^. However, because of taxonomical uncertainties when considering morphological characters alone, the identifications of some species were challenging, especially for some speciose families such as Mugilidae, Gobiidae or Eleotridae^15^. Furthermore, cryptic diversity is frequently encountered in tropical highly biodiverse regions^16,17^, and it is possible that some morphology-based species hide more than one species. In Merbok as elsewhere, a precise account of species diversity is a necessary requirement for further researches and numerous studies have highlighted the complementarity between morphological and molecular approaches to reveal biodiversity^18–20^. To date, there is no attempt to compare morphology-based results on fish diversity with genetics-based approach in this mangrove species-rich community.

Since its introduction in the past decades, DNA barcoding has emerged as the global molecular taxonomic method across fishes based on a standard molecular marker, a ~ 650 base pairs long fragment of the mitochondrial cytochrome oxidase I gene (COI)^21^. Several regional DNA barcoding studies have demonstrated its efficacy to delimitate marine fish species, for instance, in Australia^22^, South China Sea^23^, Indian Ocean^24^, and Indo-Pacific coral fishes^25^. DNA barcoding has proven to be a reliable method in detecting cryptic and potentially new fish species^26–29^, identifying larval fishes^30–33^, or tracing back food origins^34^.

In this study, we assemble a reference library of DNA barcodes of 350 fish individuals from Merbok Estuary and its adjacent waters for the purpose to describe the fish diversity in this region in providing a complementary look at previous morphology-based results. Comprehensive species lists built on integrative taxonomy have wide applications including ecosystem health management, biodiversity monitoring and conservation, aquaculture and fishery management^35,36^. All of these uses pertain to the Merbok Estuary.

## Results

### Fish diversity

A total of 350 specimens (out of 441 collected) were successfully sequenced for the COI gene, representing 134 morphological species, 94 genera, 47 families, 17 orders, and two classes, Chondrichthyes and Actinopterygii (taxonomic list shown Table 1). Two of these species (i.e. *Cryptocentrus* sp. and *Johnius* sp.) were only identified to the generic level using morphology whereas for three other species, *Dichotomyctere* cf. *fluviatilis*, *Brachygobius* cf. *kabiliensis,* and *Cynoglossus* cf. *cynoglossus,* we used open nomenclature.

**Table 1:** List of morphology-based species from Merbok Estuary region studied through DNA barcoding with the number of specimens examined (n), the BOLD IDs of their respective COI sequences, and the museum catalogue numbers of each species.

ORDER, Family, *Species*	n	BOLD ID	MUSEUM CATALOGUE NO
**MYLIOBATIFORMES**			
**Dasyatidae**			
*Brevitrygon walga*	1	DBMR332-20	USMFC (1) 00003
*Telatrygon zugei*	1	DBMR237-19	USMFC (1) 00002
**Gymnuridae**			
*Gymnura poecilura*	1	DBMR036-19	USMFC (104) 00001
**ORECTOLOBIFORMES**			
**Hemiscylliidae**			
*Chiloscyllium indicum*	1	DBMR335-20	USMFC (114) 00001
**ANGUILLIFORMES**			
**Ophichthidae**			
*Pisodonophis cancrivorus*	1	DBMR001-19	USMFC (106) 00001
**CLUPEIFORMES**			
**Chirocentridae**			
*Chirocentrus nudus*	1	DBMR331-20	USMFC (72) 00001
**Clupeidae**			
*Anodontostoma chacunda*	1	DBMR341-20	USMFC (5) 00005
*Escualosa thoracata*	3	DBMR342-20—DBMR344-20	USMFC (5) 00003
*Sardinella albella*	2	DBMR224-19, DBMR315-19	USMFC (5) 00007, USMFC (5) 00009
**Dussumieriidae**			
*Dussumieria albulina*	3	DBMR316-19—DBMR318-19	USMFC (103) 00001
**Engraulidae**			
*Setipinna taty*	1	DBMR009-19	USMFC (82) 00047
*Stolephorus baweanensis*	1	DBMR011-19	USMFC (82) 00045
*Stolephorus mercurius*	1	DBMR314-19	USMFC (82) 00050
*Stolephorus baganensis*	7	DBMR220-19—DBMR223-19, DBMR311-19—DBMR313-19	USMFC (82) 00038, USMFC (82) 00049
*Stolephorus indicus*	1	DBMR340-20	USMFC (82) 00044
*Stolephorus tri*	1	DBMR010-19	USMFC (82) 00039
*Thryssa hamiltonii*	4	DBMR012-19—DBMR014-19, DBMR016-19	USMFC (82) 00041, USMFC (82) 00043
*Thryssa kammalensis*	4	DBMR018-19—DBMR021-19	USMFC (82) 00042, USMFC (82) 00046
*Thryssa mystax*	2	DBMR015-19, DBMR017-19	USMFC (82) 00051, USMFC (82) 00052
**Pristigasteridae**			
*Ilisha melastoma*	6	DBMR022-19—DBMR027-19	USMFC (92) 00004, USMFC (92) 00006
*Opisthopterus tardoore*	2	DBMR028-19—DBMR029-19	USMFC (92) 00007
**SILURIFORMES**			
**Ariidae**			
*Arius gagora*	3	DBMR273-19—DBMR275-19	USMFC (66) 00005
*Arius maculatus*	5	DBMR192-19 -DBMR196-19	USMFC (66) 00008
*Hexanematichthys sagor*	3	DBMR198-19—DBMR200-19	USMFC (66) 00002
*Ketengus typus*	1	DBMR201-19	USMFC (66) 00006
*Osteogeneiosus militaris*	4	DBMR202-19—DBMR204-19, DBMR345-20	USMFC (66) 00009
*Plicofollis argyropleuron*	3	DBMR205-19—DBMR207-19	USMFC (66) 00003, USMFC (66) 0004
*Plicofollis layardi*	1	DBMR272-19	USMFC (66) 00007
*Plicofollis polystaphylodon*	1	DBMR197-19	USMFC (66) 00010
**Plotosidae**			
*Plotosus canius*	1	DBMR208-19	USMFC (93) 00002
**AULOPIFORMES**			
**Synodontidae**			
*Saurida micropectoralis*	1	DBMR329-20	USMFC (51) 00003
**BATRACHOIDIFORMES**			
**Batrachoididae**			
*Allenbatrachus grunniens*	1	DBMR336-20	USMFC (102) 00002
*Batrachomoeus trispinosus*	1	DBMR002-19	USMFC (102) 00001
**GOBIIFORMES**			
**Eleotridae**			
*Butis butis*	5	DBMR072-19, DBMR300-19—DBMR301-19, DBMR322-19, DBMR323-19	USMFC (33) 00012, USMFC (33) 00003, USMFC (33) 00014
*Butis humeralis*	8	DBMR065-19—DBMR071-19, DBMR306-19	USMFC (33) 00001, USMFC (33) 00002, USMFC (33) 00004
*Butis koilomatodon*	1	DBMR302-19	USMFC (33) 00013
**Gobiidae**			
*Acentrogobius caninus*	1	DBMR082-19	USMFC (34) 00015
*Boleophthalmus boddarti*	1	DBMR083-19	USMFC (34) 00016
*Brachygobius aggregatus*	3	DBMR285-19—DBMR287-19	USMFC (34) 00020
*Exyrias puntang*	3	DBMR084-19, DBMR304-19, DBMR339-20	USMFC (34) 00018, USMFC (34) 00025
*Favonigobius gymnauchen*	2	DBMR085-19—DBMR086-19	USMFC (34) 00011, USMFC (34) 00014
*Glossogobius aureus*	6	DBMR087-19—DBMR092-19	USMFC (34) 00010, USMFC (34) 00012
*Hemigobius hoevenii*	1	DBMR296-19	USMFC (34) 00023
*Psammogobius biocellatus*	1	DBMR305-19	USMFC (34) 00026
*Pseudapocryptes elongatus*	2	DBMR093-19—DBMR094-19	USMFC (34) 00017
*Pseudogobius fulvicaudus*	3	DBMR297-19—DBMR299-19	USMFC (34) 00024
*Pseudogobius avicennia*	2	DBMR294-19—DBMR295-19	USMFC (34) 00022
*Stigmatogobius sadanundio*	5	DBMR095-19—DBMR096-19, DBMR291-19 -DBMR293-19	USMFC (34) 00019, USMFC (34) 00021
*Trypauchen vagina*	1	DBMR321-19	USMFC (34) 00027
*Trypauchen pelaeos*	1	DBMR097-19	USMFC (34) 00013
*Cryptocentrus* sp.	1	DBMR338-20	N/A
**ATHERINIFORMES**			
**Phallostethidae**			
*Neostethus lankesteri*	3	DBMR282-19—DBMR284-19	USMFC (108) 00001
**BELONIFORMES**			
**Adrianichthyidae**			
*Oryzias javanicus*	3	DBMR279-19—DBMR281-19	USMFC (101) 00002
**Belonidae**			
*Strongylura strongylura*	3	DBMR003-19—DBMR005-19	USMFC (89) 00002
**Hemiramphidae**			
*Hyporhamphus dussumieri*	1	DBMR219-19	USMFC (105) 00002
*Hyporhamphus quoyi*	4	DBMR006-19—DBMR008-19, DBMR324-19	USMFC (105) 00001, USMFC (105) 00005
**Zenarchopteridae**			
*Dermogenys sumatrana*	3	DBMR288-19—DBMR290-19	USMFC (105) 00004
**CARANGIFORMES**			
**Carangidae**			
*Alepes melanoptera*	2	DBMR043-19—DBMR044-19	USMFC (69) 00013, USMFC (69) 00014
*Atule mate*	1	DBMR045-19	USMFC (69) 00015
*Carangoides coeruleopinnatus*	3	DBMR046-19—DBMR047-19, DBMR247-19	USMFC (69) 00012
*Caranx ignobilis*	4	DBMR048-19—DBMR051-19	USMFC (69) 00019, USMFC (69) 00020
*Caranx sexfasciatus*	1	DBMR346-20	USMFC (69) 00022
*Megalaspis cordyla*	5	DBMR052-19—DBMR056-19	USMFC (69) 00009
*Scomberoides commersonnianus*	1	DBMR060-19	USMFC (69) 00016
*Scomberoides tala*	1	DBMR059-19	USMFC (69) 00018
*Scomberoides tol*	2	DBMR057-19—DBMR058-19	USMFC (69) 00017
*Selaroides leptolepis*	2	DBMR328-20, DBMR330-20	USMFC (69) 00010
*Trachinotus blochii*	2	DBMR061-19—DBMR062-19	USMFC (69) 00021
*Ulua mentalis*	1	DBMR246-19	USMFC (69) 00011
**Cynoglossidae**			
*Cynoglossus arel*	1	DBMR268-19	USMFC (84) 00007
*Cynoglossus* cf. *cynoglossus*	3	DBMR187-19, DBMR325-19, DBMR334-20	USMFC (84) 00003, USMFC (84) 00006
*Cynoglossus monopus*	4	DBMR269-19—DBMR271-19, DBMR319-19	USMFC (84) 00002, USMFC (84) 00005
*Cynoglossus bilineatus*	3	DBMR188-19—DBMR190-19	USMFC (84) 00008
*Cynoglossus oligolepis*	1	DBMR320-19	USMFC (84) 00004
**Paralichthyidae**			
*Pseudorhombus arsius*	1	DBMR327-20	USMFC (107) 00001
**Latidae**			
*Lates calcarifer*	5	DBMR102-19—DBMR106-19	USMFC (76) 00001
**Polynemidae**			
*Eleutheronema tetradactylum*	7	DBMR137-19—DBMR143-19	USMFC (68) 00005, USMFC (68) 00006
*Leptomelanosoma indicum*	1	DBMR144-19	USMFC (68) 00004
**MUGILIFORMES**			
**Mugilidae**			
*Crenimugil buchanani*	2	DBMR226-19, DBMR236-19	USMFC (81) 00006
*Crenimugil crenilabis*	3	DBMR225-19—DBMR228-19	USMFC (81) 00007
*Osteomugil perusii*	2	DBMR229-19—DBMR230-19	USMFC (81) 00003, USMFC (81) 00004
*Planiliza subviridis*	12	DBMR030-19—DBMR035-19, DBMR231-19—DBMR235-19, DBMR333-20	USMFC (81) 00001, USMFC (81) 00002, USMFC (81) 00005, USMFC (81) 00008
**PERCIFORMES**			
**Gerreidae**			
*Gerres filamentosus*	4	DBMR073-19—DBMR074-19, DBMR081-19, DBMR248-19	USMFC (91) 00005, USMFC (91) 00006
*Gerres limbatus*	4	DBMR077-19—DBMR080-19	USMFC (91) 00004
*Gerres oyena*	2	DBMR075-19—DBMR076-19	USMFC (91) 00003
**Ambassidae**			
*Ambassis vachellii*	8	DBMR238-19—DBMR245-19	USMFC (30) 00002, USMFC (30) 00003
*Ambassis interrupta*	3	DBMR040-19—DBMR042-19	USMFC (30) 00006
*Ambassis macracanthus*	5	DBMR037-19—DBMR039-19, DBMR347-20—DBMR348-20	USMFC (30) 00004, USMFC (30) 00005, USMFC (30) 00007
**Haemulidae**			
*Pomadasys kaakan*	4	DBMR098-19—DBMR101-19	USMFC (71) 00002, USMFC (71) 00003, USMFC (71) 00004, USMFC (71) 00005
**Lethrinidae**			
*Lethrinus lentjan*	3	DBMR128-19—DBMR130-19	USMFC (79) 00001
**Lutjanidae**			
*Lutjanus argentimaculatus*	1	DBMR136-19	USMFC (49) 00012
*Lutjanus johnii*	2	DBMR131-19—DBMR132-19	USMFC (49) 00007, USMFC (49) 00011
*Lutjanus russellii*	7	DBMR134-19—DBMR135-19, DBMR250-19 -DBMR253-19, DBMR133-19	USMFC (49) 00010, USMFC (49) 00006, USMFC (49) 00008, USMFC (49) 00009
**Sciaenidae**			
*Dendrophysa russelii*	1	DBMR150-19	USMFC (48) 00009
*Johnius* sp.	5	DBMR254-19—DBMR257-19, DBMR259-19	USMFC (48) 00008
*Nibea soldado*	3	DBMR151-19—DBMR153-19	USMFC (48) 00005
*Otolithes ruber*	5	DBMR154-19—DBMR158-19	USMFC (48) 00004
*Panna microdon*	1	DBMR260-19	USMFC (48) 00007
*Pennahia anea*	1	DBMR266-19	USMFC (48) 00006
*Pennahia ovata*	5	DBMR261-19—DBMR265-19	USMFC (48) 00003
**Serranidae**			
*Cephalopholis formosa*	1	DBMR278-19	USMFC (74) 00008
*Epinephelus bleekeri*	1	DBMR277-19	USMFC (74) 00009
*Epinephelus coioides*	5	DBMR159-19—DBMR163-19	USMFC (74) 00006
*Epinephelus heniochus*	1	DBMR276-19	USMFC (74) 00005
*Epinephelus sexfasciatus*	1	DBMR352-20	USMFC (74) 00007
**Sillaginidae**			
*Sillago sihama*	4	DBMR170-19—DBMR173-19	USMFC (53) 00003
**Sphyraenidae**			
*Sphyraena barracuda*	1	DBMR309-19	USMFC (62) 00006
*Sphyraena jello*	2	DBMR174-19, DBMR310-19	USMFC (62) 00004, USMFC (62) 00007
*Sphyraena qenie*	1	DBMR267-19	USMFC (62) 00005
**Platycephalidae**			
*Grammoplites scaber*	1	DBMR326-20	USMFC (56) 00006
*Platycephalus indicus*	1	DBMR191-19	USMFC (56) 00007
**Tetrarogidae**			
*Trichosomus trachinoides*	2	DBMR308-19, DBMR337-20	USMFC (115) 00001, USMFC (115) 00002
**Cichlidae**			
*Oreochromis mossambicus*	1	DBMR063-19	USMFC (47) 00005
**ACANTHURIFORMES**			
**Drepaneidae**			
*Drepane punctata*	4	DBMR064-19, DBMR349-20—DBMR351-20	USMFC (60) 00003
**Leiognathidae**			
*Deveximentum ruconius*	2	DBMR124-19—DBMR125-19	USMFC (50) 00013
*Deveximentum indicium*	2	DBMR123-19, DBMR249-19	USMFC (50) 00012, USMFC (50) 00015
*Deveximentum hanedai*	1	DBMR126-19	USMFC (50) 00014
*Eubleekeria jonesi*	1	DBMR107-19	USMFC (50) 00016
*Leiognathus brevirostris*	3	DBMR108-19—DBMR110-19	USMFC (50) 00011
*Leiognathus equula*	3	DBMR111-19—DBMR113-19	USMFC (50) 00017
*Nuchequula gerreoides*	9	DBMR114-19—DBMR122-19	USMFC (50) 00010, USMFC (50) 00018
**Scatophagidae**			
*Scatophagus argus*	5	DBMR145-19—DBMR149-19	USMFC (109) 00001
**Siganidae**			
*Siganus fuscescens*	1	DBMR164-19	USMFC (67) 00001
*Siganus javus*	5	DBMR165-19—DBMR169-19	USMFC (67) 00002
**SCOMBRIFORMES**			
**Stromatidae**			
*Pampus argenteus*	6	DBMR175-19—DBMR180-19	USMFC (110) 00001, USMFC (110) 00002
**Trichiuridae**			
*Lepturacanthus savala*	3	DBMR184-19—DBMR186-19	USMFC (73) 00001
**CENTRARCHIFORMES**			
**Terapontidae**			
*Terapon jarbua*	2	DBMR182-19, DBMR307-19	USMFC (88) 00004, USMFC (88) 00006
*Terapon theraps*	2	DBMR181-19, DBMR183-19	USMFC (88) 00003, USMFC (88) 00005
**TETRAODONTIFORMES**			
**Tetraodontidae**			
*Arothron reticularis*	1	DBMR209-19	USMFC (40) 000010
*Dichotomyctere* cf. *fluviatilis*	1	DBMR210-19	USMFC (40) 00006
*Dichotomyctere nigroviridis*	1	DBMR303-19	USMFC (40) 00011
*Lagocephalus lunaris*	6	DBMR211-19—DBMR216-19	USMFC (40) 00004, USMFC (40) 00005, USMFC (40) 00007, USMFC (40) 00008
*Takifugu oblongus*	1	DBMR217-19	USMFC (40) 00009
**Triacanthidae**			
*Triacanthus nieuhofii*	1	DBMR218-19	USMFC (61) 00002, USMFC (61) 00003

**Figure 2:**
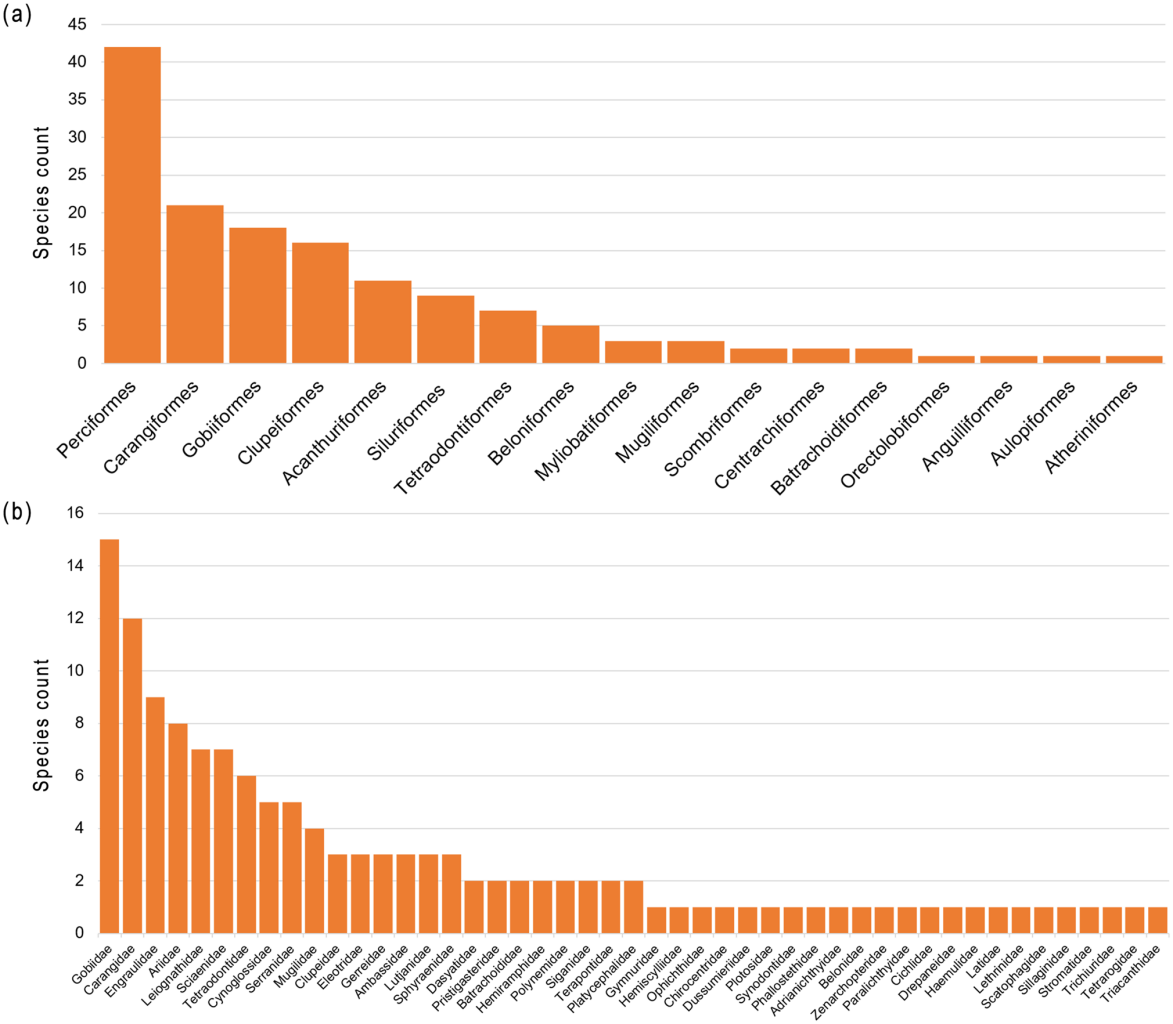
Species count rankings according to (**a**) orders and (**b**) families recorded in this study.

The most diverse orders were Perciformes (42 species representing 31.1% of the total number of species), followed by Carangiformes (21 species, 15.5%), Gobiiformes (18 species, 13.3%), and Clupeiformes (16 species, 11.9%) (Fig. 2a). At the family level, Gobiidae has the highest species richness with 15 species (11.1%), followed by Carangidae (12 species, 8.9%), Engraulidae (9 species, 6.7%), and Ariidae (8 species, 5.9%) (Fig. 2b). The three most diverse genera were the anchovy genus *Stolephorus*, the flatfish genus *Cynoglossus* with five species each, followed by the grouper genus *Epinephelus* with four species. According to the International Union for Conservation of Nature (IUCN) Red List, five species are “Near Threatened” (four Chondrichthyes: *Telatrygon zugei*, *Brevitrygon walga*, *Gymnura poecilura*, *Chiloscyllium indicum* and one actinopterygian: *Arius gagora*) whereas others are listed as “least concern” or “data deficient”. One recorded species is an alien invasive species (AIS), *Oreochromis mossambicus* (the Mozambique tilapia) from the African region^37^.

### DNA-based delimitation

Sequence length for all 350 generated barcodes was longer than 600 bp with no indels or stop codon detected. The nucleotide composition showed a mean percentage of 18.32% (G), 27.97% (C), 24.07% (A), and 30.7% (T). More than half of the species (56%, 76 species) were represented by multiple specimens while 59 species were represented by a single specimen (Table 1). Mean number of specimens per species was 2.59. Increment in the K2P genetic divergence was directly related to the hierarchical taxonomic relationship: within species mean divergence = 0.85% (SE = 0.01), within congeners mean divergence = 16.7%, (SE = 0.01) and within families mean divergence = 18.17% (SE = 0) (Table 2).

**Table 2:** K2P divergence values from 350 analysed specimens with increasing taxonomic levels.

Category	n	Taxa	Comparisons	Minimum (%)	Mean (%)	Maximum (%)	SE (%)
Within species	285	75	532	0	0.85	16.66	0.01
Within genus	169	24	427	1.11	16.7	23.59	0.01
Within family	207	16	1197	0.16	18.17	26.02	0

*SE* standard error.

**Figure 3:**
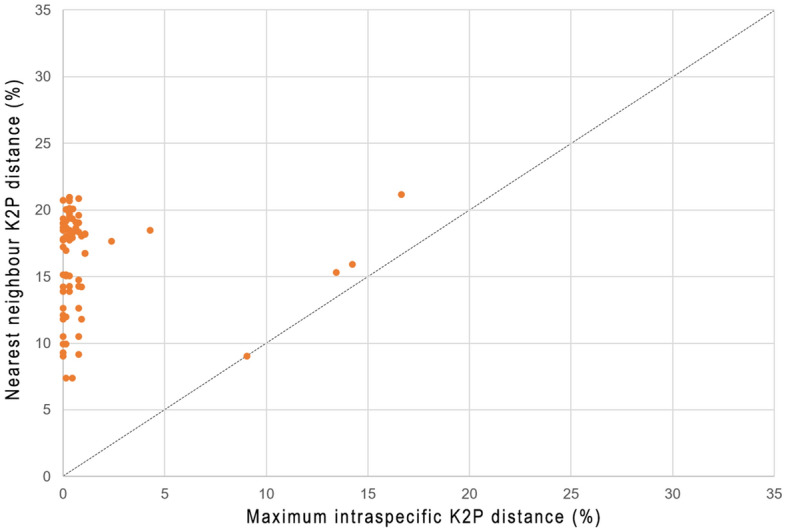
Scatterplot of maximum intraspecific K2P distances vs. the nearest neighbour K2P distances.

Deep intraspecific K2P divergences, exceeded the standard threshold distance of 2%^21,38^, were observed in seven species: *Eleutheronema tetradactylum* (16.66%), *Osteomugil perusii* (14.24%), *Planiliza subviridis* (13.44%), *Deveximentum indicium* (9.05%), *Lagocephalus lunaris* (5.62%), *Gerres oyena* (4.29%) and *Lutjanus russellii* (4.12%) (Table 3). Barcoding gap analysis demonstrated that almost all species represented by multiple sequences are supported by a barcode gap (Fig. 3). Notably, only one species, *D. indicium*, had its maximum intraspecific distance (9.05%) similar to its nearest neighbour distance (9.04%).

**Table 3:** List of morphological species comprising two MOTUs (= BINs) or sharing one MOTU. The summary statistics include the BIN of each MOTU, their maximum intraspecific distance and distance to the nearest neighbour (i.e. minimum interspecific distance).

Species/MOTUs	Max. intraspecific distance (%)	Nearest neighbour distance (%)
**Species comprising two MOTUs**		
*Deveximentum indicium*	9.05	9.04
BOLD:ADZ6313	0	9.06
BOLD:AAF1238	0	8.41
*Eleutheronema tetradactylum*	16.66	19.40
BOLD:AAB8457	0	14.44
BOLD:AAB8458	0	14.44
*Lagocephalus lunaris*	5.62	17.76
BOLD:AAF8798	0	5.07
BOLD:ADL4007	0.31	5.07
*Gerres oyena*	4.29	18.49
BOLD:AAC1288	0	4.15
BOLD:AAC1290	0	4.15
*Lutjanus russellii*	4.12	14.24
BOLD:AAB2905	0.31	3.69
BOLD:AAB2904	0	3.69
*Osteomugil perusii*	14.24	15.90
BOLD:AAG3686	0.00	12.75
BOLD:AAW7354	0.00	12.75
*Planiliza subviridis*	13.44	15.30
BOLD:ABU7210	0.92	11.98
BOLD:ACC0823	0	11.98
**Pairs of species sharing one MOTU**		
*Alepes melanoptera*	0	0.16
*Caranx sexfasciatus*	0	0.16
BOLD:AAB5775	0.16	10.45
*Dichotomyctere* cf. *fluviatilis*	0	1.11
*Dichotomyctere nigroviridis*	0	1.11
BOLD:AAF2344	1.02	11.67

**Figure 4:**
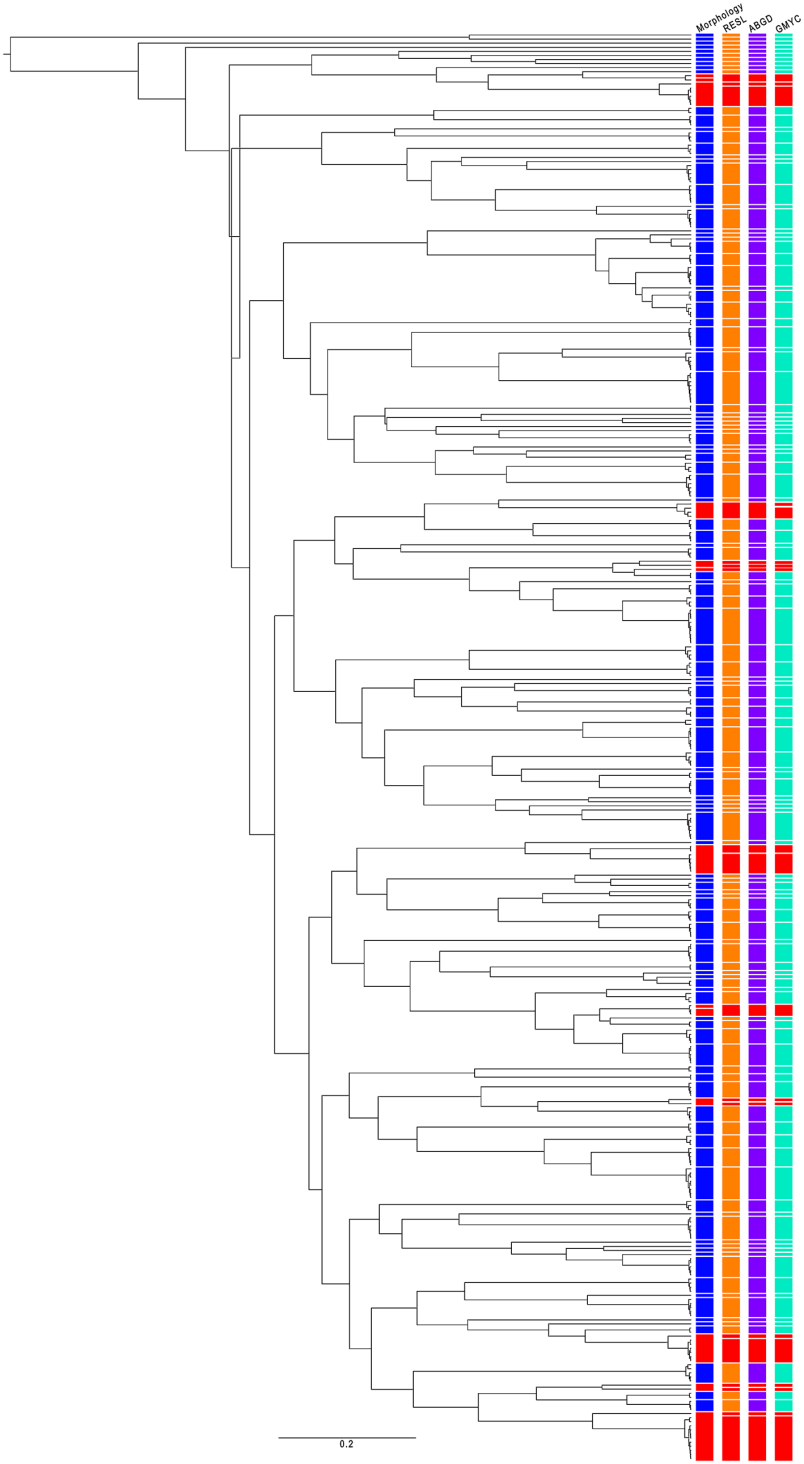
Bayesian Inference gene tree based on the 350 DNA barcodes with delineated MOTUs. Colour bars indicate (from left to right): morphological species (blue), MOTUs delineated by RESL (orange), ABGD (purple), and GMYC (green). Red bars indicate discrepancies among the different schemes (either morphology-genetics discrepancies or genetics-genetics discrepancies.

Both Bayesian Inference (BI) (Fig. 4) and Maximum Likelihood (ML) (Figure S1) trees were fully resolved exhibiting minimal differences in topologies. Node-supports in the BI tree were overall higher than in ML tree leading us to use the BI tree to visualize our Molecular Operational Taxonomic Unit (MOTU) delimitation results (Fig. 4). The three MOTU delimitation analyses (using RESL, ABGD and GMYC methods) yielded moderately variable numbers of MOTUs, although always higher than our initial 134 morphology-based species. The RESL analysis revealed 139 MOTUs assigned to dedicated BINs. The ABGD analysis identified the same 139 MOTUs (*P* = 0.0010–0.0599) within the initial partition for all substitution models (Table S2). The single-threshold GMYC analysis recognised 140 MOTUs that were taxonomically concordant with those obtained with the other two analyses except for one species, *Hyporhamphus quoyi*, that is partitioned into two MOTUs. All incongruences between MOTUs and morphology-based species delimitation are highlighted in Fig. 4 (red bars) and detailed in Table 3. In seven (eight with GMYC) cases, two MOTUs were delimitated within one morphology-based species (see above the case of *Hyporhamphus quoyi* with GMYC). In two occasions, we found two of our morphology-based species sharing the same MOTU: *Alepes melanoptera* and *Caranx sexfasciatus* (BIN “BOLD:AAB5775”) and *Dichotomyctere nigroviridis* and *Dichotomyctere* cf. *fluviatilis* (BIN “BOLD:AAF2344”) (Table 3). Within each of these two species-pairs, interspecific genetic divergence was < 2% resulting in the recognition of only one MOTU.

## Discussion

### Species delimitation

One of the premises of DNA barcoding is the detection of the so-called “barcode gap”, which can be estimated in comparing the maximum intraspecific distance with the minimum interspecific distance (also known as the nearest neighbour genetic distance)^39^. The presence of a gap within a morphological species is good evidence for species-level cryptic diversity^40^. However, the absence of gap between two morphological species is indicative either that they are different forms within one species or of shared ancestral polymorphism and/or hybridization followed by introgression between these two species. In this case, a multi-gene (i.e. genomic) approach will help to determine the reciprocal taxonomic status of the two morphological species.

Employing multiple “automatic species delimitation” methods and schemes in clustering the generated DNA barcodes provide an efficient approach in identifying putative species (= MOTUs). Even though these methods may have individual pitfalls, especially in analysing singletons, they can yield a robust outcome when combined^41^. Despites different analytical assumptions supporting each method, all three methods yielded similar results: RESL and ABGD analyses delimitated each 139 MOTUs in our dataset whereas the GMYC analysis identified 140 MOTUs. These results demonstrate a robust pattern of MOTUs in our dataset; even the GMYC method which is known to overestimate MOTUs counts compared to other methods^42^, delimitated only one additional MOTU. Because both RESL and ABGD analyses had closer correspondence to the number of species defined by morphological identification, we based our discussion on species account on these two methods.

Our results show that DNA barcoding (using COI gene) and morphology-based approach converge on the delimitation of 123 species (about 90% of the examined species) in Merbok Estuary region. DNA barcoding approach further revealed possible cryptic diversity within six species whereas it did not detect significant difference between two pairs of morphological species. Such results call for further taxonomic studies.

The mean conspecific K2P divergence (0.85%) was 20-fold lower than the mean congeneric divergence (16.7%). This increase in genetic divergence with increment in taxonomic levels is logical^35^. However, both mean genetic estimates are higher than those previously recorded in other regions. Most molecular assessment of marine fishes displayed conspecific divergence within the range of 0.25–0.39% whereas congeneric divergence were within the range of 4.56–9.93%^22–24,36,43^, but^25^ found similar pattern of high average conspecific and congeneric divergence within the Indo-Pacific coral reef fishes (1.06% and 15.34%, respectively).

### Taxonomic conundrum

We found that seven of our morphological species comprised two MOTUs: *Eleutheronema tetradactylum* (inter-MOTU COI-based genetic distance = 16.66%), *Osteomugil perusii* (14.24%), *Planiliza subviridis* (13.44%), *Deveximentum indicium* (9.05%), *Lagocephalus lunaris* (5.62%), *Gerres oyena* (4.29%) and *Lutjanus russellii* (4.12%). Such high intraspecific genetic divergence suggests either misidentification or the presence of morphologically cryptic species^25,44^. The first possibility is unlikely because the morphological examination of incriminated specimens, based on existing keys, seems consistent. Therefore, such genetic variability may more likely be the signal of hidden diversity. Large genetic differentiation has been reported in *E. tetradactylum* (family Polynemidae) among allopatric populations within the Indian Ocean^45^. Our results are consistent with^45^, further indicating that differentiation in this lineage is not only allopatrically but, also, sympatrically distributed. Recent molecular taxonomic studies on the family Mugilidae in which are included *O. perusii* and *P. subviridis*, evidenced a very high level of cryptic diversity in the Indo-West Pacific region^46,47^. Several mullet species (*P. subviridis* and *O. perusii* are among them) are, actually, each, a complex of several morphologically similar species for which extensive taxonomic revisions are needed. The taxonomy of *D. indicium* (family Leiognathidae) is still in flux with continual descriptions of new species in several genera, including *Deveximentum*^48^. The taxonomy of the genus *Lagocephalus* is difficult and the current identification key is likely incomplete making the delimitating between species challenging. Our results indicate the presence of two sympatric species under *D. indicium* in Merbok Estuary. *Gerres oyena* (family Gerreidae) and *L. russellii* (family Lutjanidae) exhibit intraspecific differentiation of lower magnitude than those observed for the first five species discussed above, although still well above the threshold of 2%. *Lutjanus russellii* natively occurs in this region^49^ but it is also farmed in Merbok estuary. Aquaculture activities regularly import non-native seeds from various sources, with no or poor records of origins. The divergence observed within this species (4.12%) could be the consequence of the presence of both native and alien (escaped from aquaculture farms) individuals in Merbok estuary^15^.

Two cases of shared MOTUs between species were detected involving the pairs *Alepes melanoptera* and *Caranx sexfasciatus* (BOLD:AAB5775), and *Dichotomyctere nigroviridis* and *Dichotomyctere* cf. *fluviatilis* (BOLD:AAF2344). The first case is striking because *A. melanoptera* and *C. sexfasciatus* are morphologically easily distinguishable (specimens are housed in the USMFC collections and available for morphological verification) and the two COI sequences (one from each of these two species) are only slightly different, which seems to exclude the possibility of a contamination. This observation warrants future investigation based on more specimens.

The second case is interesting because the marking patterns of the specimens of *D. nigroviridis* and *D.* cf. *fluviatilis* are distinctly different^15^. However, the genetic distance between these two species is only 1.1%. We hypothesize that, in this case, the COI-based genetic differentiation (< 2%) between *D. nigroviridis* and *D.* cf. *fluviatilis* does not reflect their actual taxonomical status. Recent hybridisation among these two closely related species and incomplete lineage sorting of a recent, on-going speciation event could account for this observation^50^. Guimarães-Costa et al.^51^ who studied the fish diversity in the Parnaíba Delta, also suggested that the rate of molecular variation does not necessary accompany recent (sympatric) speciation event that lead to morphological differentiation.

### Towards the establishment of a comprehensive DNA barcoding library of the fish community of Merbok Estuary

Precise identification of organisms is a prerequisite for assessing the biological and ecological status of an ecosystem. The current study illustrates yet another example of the complementarity of the morphological and molecular techniques to achieve this goal. DNA barcoding offers a quick and easy approach in aquatic diversity assessment and requires minimal expertise in conventional taxonomy^52,53^. Comprehensive DNA barcode reference library is crucial in any biodiversity assessment for providing selective autecological and biogeographic information for comparative analysis with previous assessment. Even though DNA databases like BOLD^54^ and GenBank^55^ are publicly available, a localised taxon-specific reference library is synoptically important as it is easier to curate and is a more practical reference for a focused site.

Our DNA barcodes reference library associated with voucher collections previously established^15^ can be used for further biological evaluation and biomonitoring effort in Merbok Estuary and nearby regions. Future research endeavours to assess ecosystem health status in which a reference DNA barcoding library is needed, such as COI-based environmental DNA (eDNA) surveys or metabarcoding assays, can use this database. The barcode data generated in this study will contribute to the local as well as regional conservation efforts of fish diversity. Notwithstanding, to improve the resolution of the taxonomic coverage of the mangrove-associated of the fish community of Merbok Estuary, the number of DNA barcodes for the singleton specimens and also the not-yet examined species should be increased through more sampling and increased number of sites within the estuary and around.

Of the 134 species examined in this study, 61 species (~46%) were identified with high commercial value^56^. Protection planning and proper fishery management of these species are vital. Furthermore, we manage to barcode an invasive species—the Mozambique tilapia, *Oreochromis mossambicus*; its monitoring should be conducted either using traditional methods or eDNA methods.

We DNA barcoded a rich and diverse mangrove-associated fish community. Of the 134 species initially identified based on morphology, barcodes of 123 species support their validity. We found hidden diversity within seven species whereas the divergences between two pairs of valid species are below the interspecific threshold standard calling for further taxonomic studies. The comparison with previous species lists in and around this region^49^ shows that our taxonomic coverage in Merbok Estuary is certainly not complete, although the degree of incompleteness is unknown. Further researches are needed to expand the results of this study, especially towards small, elusive, transient and non-commmercial fish species. The establishment of a local DNA barcoding reference library is an essential step for future studies of fisheries, conservation and ecological management of this important site.

## Methods

### Ethics statement

This project was conducted according to the relevant national and international guidelines and did not involve any endangered or protected fish species. All fish specimens were either collected from the local fishermen, caught using non-invasive fishing gear by the authors, or bought from the local market. This study was carried out following the recommendations and approval by the Universiti Sains Malaysia Animal Ethics Committee.

### Sample collection

A total of 441 specimens were sampled between December 2018 to October 2019 at multiple locations along the Merbok Estuary and its vicinity (Fig. 1). Specimens were collected either from local fishermen (who use the barrier-net method locally called ‘pompang’), direct sampling by dip-net or bought from the major fish landing site (Kuala Muda Whispering Market). All specimens were caught within Merbok River and its adjacent waters. Samples collected from the fish landing site were retrieved from fishing vessels that operate within Zone A (from the shoreline up to 5 nautical miles) and Zone B (from 5 to 12 nautical miles)^57^. Information on the sampling localities (geographical coordinates) is shown in Table S1. Other collection data—dates, taxonomy and details of voucher specimens can be retrieved from the online project datasheet implemented in BOLD with project code—DBMR.

### Sample processing and morphological identification

A fin clip from each fresh specimen was taken and stored in 90% ethanol. Voucher specimens were fixed in 10% formalin for at least one week and then transferred into 70% ethanol for long term storage. All specimens were catalogued and deposited at the Museum of Biodiversity, Universiti Sains Malaysia.

Morphology-based species identifications and nomenclature follow^15^ with few reidentifications: *Pseudogobius avicennia* (museum number: USMFC (34) 00022; identified as *Pseudogobius olorum* in^15^), *Trypauchen vagina* (USMFC (34) 00027; *Trypauchen pelaeos* in^15^), *Trypauchen pelaeos* (USMFC (34) 00013; *Trypauchen vagina* in^15^), *Cynoglossus bilineatus* (USMFC (84) 00008; *Cynoglossus lingua* in^15^), *Cynoglossus monopus* (USMFC (84) 00002, 00005; *Cynoglossus cynoglossus* in^15^), *Cynoglossus* cf. *cynoglossus* (USMFC (84) 00003, 00006; *Cynoglossus puncticeps* in^15^), *Pseudorhombus arsius* (USMFC (107) 00001; *Pseudorhombus elevatus* in^15^), *Stolephorus baganensis* (USMFC (82) 00038, 00049; *Stolephorus dubiosus* in^15^). We were unable to unequivocally assigned few specimens to a valid described species using available keys. In these cases, we used either “sp.” or “cf.”.

We did not barcode five species listed in^15^: *Sardinella gibbosa*, *Zenarchopterus buffonis*, *Gerres macracanthus*, *Drepane longimana*, and *Johnius belangerii*, but we sequenced one specimen of *Cryptocentrus* sp., which was not listed in^15^. A total of 134 morphological species were considered in this study (Table 1).

### Laboratory analyses

Genomic DNA was extracted using DNeasy Blood & Tissue kit (Qiagen, Germany) following the given protocol of animal tissue DNA extraction. The purity and concentration of the isolated DNA were measured using a microvolume UV spectrophotometer (Quawell Q300, Quawell, CA) and stored at − 20 °C until further use. An approximately 650 bp fragment of the mitochondrial COI gene region was amplified using the combinations of the following primers previously designed by^22^:

- FishF1-5’TCAACCAACCACAAAGACATTGGCAC-3’,
- FishF2-5’-TCGACTAATCATAAAGATATCGGCAC-3’,
- FishR1-5’-TAGACTTCTGGGTGGCCAAAGAATCA-3’ and
- FishR2-5’-ACTTCAGGGTGACCGAAGAATCAGAA-3’.

Each sample was amplified in a final volume of 25 µL, containing 5.5 µL of 5x MyTaq™ Reaction Buffer Red (Bioline GmbH, Germany), 0.5 µL of each primer (100 ng/µL), 0.25 µL 5U Taq polymerase (iNtRON Biotechnology Inc., Korea), 2.5 µL of genomic DNA (50 ng/µL) and adequate nuclease-free water to complete the final reaction volume. Each amplification set was performed with the inclusion of a negative control (no template DNA) with thermal cycling conditions as follows: initial denaturation at 94 °C for 4 min; followed by 35 cycles of denaturation at 94 °C for 30 s, annealing at 48 °C for 50  s, and extension at 72 °C for 1 min; then a final extension at 72 °C for 10 min. The PCR products were then fractioned by 2% gel electrophoresis to check for successful amplification. All positive amplifications were then sent for purification and sequencing to Apical Scientific Sdn. Bhd. (Selangor, Malaysia) operating the ABI PRISM 3730XL automated sequencer and the ABI PRISM BigDye terminator cycle sequencing kit v3.1 (Applied Biosystems, Foster City, CA). Bidirectional sequencing was employed to decrease the probability of sequencing errors.

### Data analyses

Each generated chromatogram was manually screened prior to DNA alignment in MEGA X^58^. The sequences were proofread and independently aligned and then inspected for deletions, insertions and stop codons using the same software.

A total of 350 COI sequences were determined in this study. To assess the taxon discrimination between all specimens, pairwise genetic distances were calculated within and between species, genera, and families based on the Kimura 2-parameter (K2P) distance model^59^ using the analytical tools available in the BOLD system platform. To depict a graphical representation of the genetic relationships of the sequences, Bayesian Inference (BI) and Maximum Likelihood (ML) analyses were run in BEAST 2^60^ and raxmlGUI 2.0^61^ program, respectively. The GTR+I+G substitution model was determined as the best one in PartitionFinder 2^62^, as implemented in the CIPRES portal^63^. The BI tree was constructed with the GTR+I+G substitution model, empirical base frequencies with four gamma categories, employing a relaxed lognormal clock and the birth-death model. Two Markov Chain Monte Carlo (MCMC) chains of 40 million were run independently, sampled every 1000 generations and the first 20% were discarded as burn-in. Both run performances were then assessed for convergence (ESS > 200) using Tracer 1.7.1 and combined using LogCombiner 2.4.8 before the final tree was constructed using TreeAnnotator 2.4.7, within the BEAST 2 package^60^. The ML tree was also built based on the GTR+I+G model with 1000 nonparametric bootstrap replicates. Both constructed trees were then viewed and edited in FigTree 1.4.4^64^.

Three different sequence-based methods were used to delimit the Molecular Operational Taxonomic Units (MOTUs) from the analysed sequences—(1) Refined Single Linkage (RESL), (2) Automatic Barcode Gap Discovery (ABGD), and (3) Generalized Mixed Yule Coalescent (GMYC). The first analysis was done within the BOLD platform using the RESL algorithm^65^ to assign sequences to a dedicated Barcode Index Numbers (BINs which are MOTUs). Next, the ABGD^39^ analysis was run at the webserver (https://bioinfo.mnhn.fr/abi/public/abgd/abgdweb.html) to census divergence within the analysed dataset for species delimitation. The ABGD analysis was run with the following settings: relative gap width X=1.0, intraspecific divergence (*P*) values range from 0.001 to 0.0059 for all the distance metrics, while all other parameter values were kept as default. Finally, the GMYC method^66^ was employed with the fully resolved, BI ultrametric tree using only unique haplotypes (see above for the reconstruction method). The haplotype dataset used in the GMYC analysis was built in collapsing all 350 individual COI sequences into 258 unique haplotype sequences using ALTER^67^. A single-threshold GMYC analysis was run in RStudio^68^ with the ‘splits’ package^69^.

## Supplementary Information

Supplementary Information.

## Data Availability

All the COI sequences determined in this study have been uploaded in BOLD^54^ under the DBMR project (dx.doi.org/10.5883/DS-SRDBMR) and deposited in GenBank^55^ (Accession nos. MW498499—MW498843).
