# Supplementary material for: DNA-based taxonomy of a mangrove-associated community of fishes in Southeast Asia
Source: Sci Rep. 2021 Sep 7;11:17800. doi: 10.1038/s41598-021-97324-1 (PMC8423740; doi:10.1038/s41598-021-97324-1)
Supplement: Supplementary file 1 — Supplementary Information. [file 41598_2021_97324_MOESM1_ESM.docx]

**Supplementary information**

**
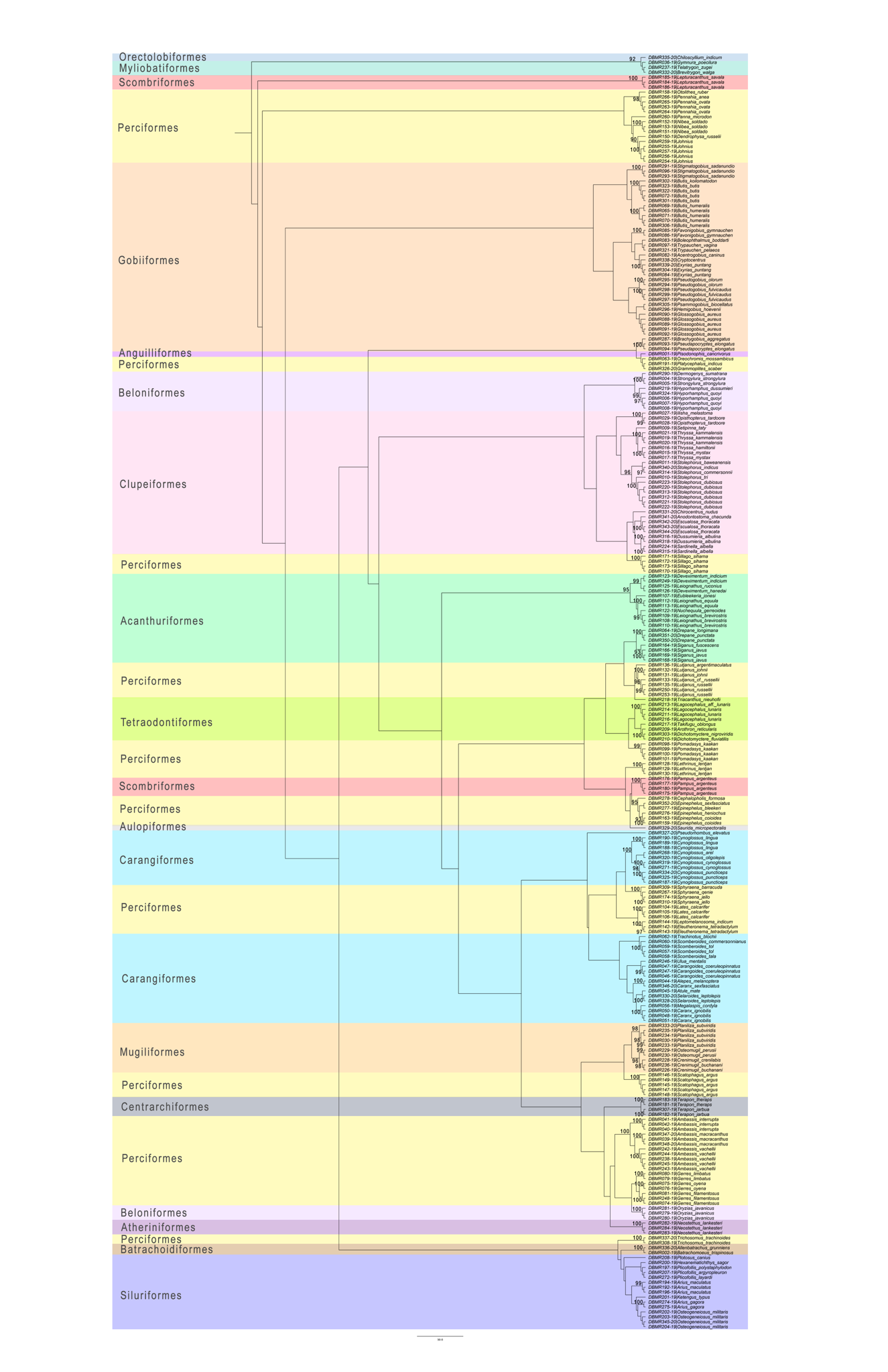
**

**Figure S1.** Maximum Likelihood (1000 replicates) of the barcoded specimens with the exclusion of identical haplotypes. Only ML bootstrap values over 90% were shown.

**Table S1.** Geographical coordinates of the sampling localities in the Merbok River estuary and its adjacent waters.

| **Site** | **Locality** | **Geographic coordinates** | |
| --- | --- | --- | --- |
|  |  | **N** | **E** |
| 1 | Kuala Muda Whispering Market | 5.578° | 100.341° |
| 2 | Pompang Sungai Merbok | 5.664° | 100.381° |
| 3 | Pompang Batu Lintang (A) | 5.624° | 100.395° |
| 4 | Pompang Batu Lintang (B) | 5.625° | 100.394° |
| 5 | Semeling Bridge | 5.680° | 100.470° |

**Table S2:** Number of OTUs identified from 350 sequences through Automatic Barcode Gap Discovery (ABGD) using multiple substitution model.

| Substitution Model | Relative gap width (X) | Partitions | Prior intraspecific divergence (P) | | | | | | | | |
| --- | --- | --- | --- | --- | --- | --- | --- | --- | --- | --- | --- |
|  |  |  | 0.0010 | 0.0017 | 0.0028 | 0.0046 | 0.0077 | 0.0219 | 0.0215 | 0.0359 | 0.0599 |
| Jukes Cantor (JC) | 1.0 | Initial | 139 | 139 | 139 | 139 | 139 | 139 | 139 | 139 | 139 |
|  |  | Recursive | 164 | 141 | 141 | 140 | 139 | 139 | 139 | 139 | 139 |
| Kimura-2 parameter (K2P) | 1.0 | Initial | 139 | 139 | 139 | 139 | 139 | 139 | 139 | 139 | 139 |
|  |  | Recursive | 164 | 141 | 141 | 140 | 139 | 139 | 139 | 139 | 139 |
| p-distance (simple) | 1.0 | Initial | 139 | 139 | 139 | 139 | 139 | 139 | 139 | 139 | 139 |
|  |  | Recursive | 139 | 139 | 139 | 139 | 139 | 139 | 139 | 139 | 139 |
